# Bronchial mucosal IFN-α/β and pattern recognition receptor expression in patients with experimental rhinovirus-induced asthma exacerbations

**DOI:** 10.1016/j.jaci.2018.04.003

**Published:** 2019-01

**Authors:** Jie Zhu, Simon D. Message, Patrick Mallia, Tatiana Kebadze, Marco Contoli, Christine K. Ward, Elliot S. Barnathan, Mary Ann Mascelli, Onn M. Kon, Alberto Papi, Luminita A. Stanciu, Michael R. Edwards, Peter K. Jeffery, Sebastian L. Johnston

**Affiliations:** a Airway Disease Infection, National Heart and Lung Institute, Medical Research Council and Asthma UK Centre in Allergic Mechanisms of Asthma, Imperial College London, London, United Kingdom; b Imperial College Healthcare NHS Trust, London, United Kingdom; c Research Centre on Asthma and COPD, University of Ferrara, Ferrara, Italy; d Centocor, Malvern, Pa

**Keywords:** Asthma exacerbation, rhinovirus infection, type I interferon, pattern recognition receptors, AHR, Airway hyperresponsiveness, BAL, Bronchoalveolar lavage, HRP, Horseradish peroxidase, MDA5, Melanoma differentiation–associated gene 5, PEF, Peak expiratory flow, PRR, Pattern recognition receptor, RIG-I, Retinoic acid–inducible protein I, RV16, Rhinovirus 16, TLR, Toll-like receptor

## Abstract

**Background:**

The innate immune system senses viral infection through pattern recognition receptors (PRRs), leading to type I interferon production. The role of type I interferon and PPRs in rhinovirus-induced asthma exacerbations *in vivo* are uncertain.

**Objectives:**

We sought to compare bronchial mucosal type I interferon and PRR expression at baseline and after rhinovirus infection in atopic asthmatic patients and control subjects.

**Methods:**

Immunohistochemistry was used to detect expression of IFN-α, IFN-β, and the PRRs: Toll-like receptor 3, melanoma differentiation–associated gene 5, and retinoic acid–inducible protein I in bronchial biopsy specimens from 10 atopic asthmatic patients and 15 nonasthmatic nonatopic control subjects at baseline and on day 4 and 6 weeks after rhinovirus infection.

**Results:**

We observed IFN-α/β deficiency in the bronchial epithelium at 3 time points in asthmatic patients *in vivo*. Lower epithelial IFN-α/β expression was related to greater viral load, worse airway symptoms, airway hyperresponsiveness, and reductions in lung function during rhinovirus infection. We found lower frequencies of bronchial subepithelial monocytes/macrophages expressing IFN-α/β in asthmatic patients during infection. Interferon deficiency at baseline was not accompanied by deficient PRR expression in asthmatic patients. Both epithelial and subepithelial PRR expression were induced during rhinovirus infection. Rhinovirus infection–increased numbers of subepithelial interferon/PRR-expressing inflammatory cells were related to greater viral load, airway hyperresponsiveness, and reductions in lung function.

**Conclusions:**

Bronchial epithelial IFN-α/β expression and numbers of subepithelial IFN-α/β–expressing monocytes/macrophages during infection were both deficient in asthmatic patients. Lower epithelial IFN-α/β expression was associated with adverse clinical outcomes after rhinovirus infection *in vivo*. Increases in numbers of subepithelial cells expressing interferon/PRRs during infection were also related to greater viral load/illness severity.

Rhinoviruses are the major cause of asthma exacerbations.^1^, ^2^, ^3^ Experimental rhinovirus infection in asthmatic patients is associated with augmented responses to allergen challenge,^4^, ^5^ greater upper and lower respiratory tract symptoms, increases in airway hyperresponsiveness (AHR),^6^, ^7^, ^8^ reductions in peak expiratory flow (PEF),^7^, ^9^ and FEV_1_.^10^ Rhinovirus infection in asthmatic patients also increases bronchoalveolar lavage (BAL) fluid eosinophil counts,^7^ induces infiltration of neutrophils and monocytes/macrophages in the airway mucosa,^11^ and enhances T_H_2 cytokine responses.^6^, ^7^

Type I (α/β) interferons play essential roles in innate and adaptive antiviral immune responses. Deficiencies in induction of interferons by *ex vivo* virus-infected bronchial epithelial cells and BAL fluid cells in asthmatic patients has been reported in several,^12^, ^13^, ^14^, ^15^, ^16^ but not all,^17^, ^18^ studies. The relationships of type I interferons with rhinovirus-induced asthma exacerbation pathogenesis and rhinovirus infection–induced innate inflammatory responses remains uncertain.

The innate immune system senses viral infection and triggers antiviral responses by recognizing viral components through pattern recognition receptors (PRRs),^19^, ^20^ including Toll-like receptor (TLR) 3,^21^, ^22^ melanoma differentiation–associated gene 5 (MDA5), and retinoic acid–inducible protein I (RIG-I).^23^, ^24^, ^25^ These PRRs are implicated specifically in interferon induction by rhinovirus infection.^26^ Impaired baseline or rhinovirus-induced PRR expression could be involved in deficient interferon production in asthmatic patients.

Therefore we tested the hypothesis that protein expression of IFN-α and IFN-β and TLR3, MDA5, and RIG-I is impaired in the epithelium and subepithelial inflammatory cells of the bronchial mucosa in asthmatic patients at baseline and after experimental rhinovirus infection. Moreover, because *ex vivo* interferon deficiency has been related to IgE levels,^27^, ^28^ we determined whether bronchial epithelial interferon staining is related to serum IgE levels.

We also examined the association of interferon/PRR expression with 8 parameters representing the most important clinical illness severity measures assessed during these rhinovirus-induced asthma exacerbations^7^, ^11^: viral load (peak nasal lavage: day 3 sputum and day 4 BAL), clinical symptoms (total cold and total chest scores), AHR (day 6 PC_10_ histamine), and airflow obstruction (maximal decrease in PEF and in FEV_1_). Some of the findings from this study have been published in abstract form.^29^

## Methods

### Subjects

We examined bronchial biopsy specimens from 10 atopic asthmatic patients (all with mild asthma taking only inhaled short-acting β_2_ agonists) and 15 nonasthmatic nonatopic control subjects reported previously (Table I and see Table E1 in this article's Online Repository at www.jacionline.org for clinical details).^7^, ^11^ Subjects were recruited from Imperial College London Healthcare NHS Trust (St Mary's Hospital) between January 2001 and March 2004. All subjects provided written informed consent, and the study was approved by St Mary's NHS Trust Research Ethics Committee (99/BA/345). The study was conducted in accordance with the amended Declaration of Helsinki.

**Table I:** Baseline demographic data

Subjects	No.	Sex (male/female)	Age (y)∗	Skin pick test total scores∗	Serum IgE (U/mL)∗	FEV_1_ (% predicted)∗	Histamine PC_20_ (mg/mL)∗
Control subjects	15	8/7	27 ± 2.3	0 ± 0	27 ± 8.0	104 ± 3.3	20 ± 2.5
Asthmatic patients	10	2/8	23 ± 1.4	12 ± 2.5†	241 ± 49.8†	106 ± 4.4	2.7 ± 0.7†

∗ Values are means ± SEMs.

† *P* < .001 versus control subjects (Student *t* test).

### Experimental infection with rhinovirus 16

Subjects were administered a 10,000 tissue culture infective dose 50% of rhinovirus 16 (RV16) on day 0 by means of nasal spray.^7^ All subjects were confirmed infected by using standard virologic methods.^7^

### Bronchoscopy and clinical data

Bronchoscopies were performed in the endoscopy unit at St Mary's Hospital with a Keymed P100 bronchoscope (Olympus, Southend-on-Sea, United Kingdom).^7^ Bronchial biopsy specimens were taken approximately 14 days before infection (baseline) on day 4 and 6 weeks after virus inoculation. Symptoms and lung function were assessed at baseline and regularly during the infection period, AHR was assessed at baseline and day 6 after infection, and sputum and BAL viral loads were assessed on days 3 and 4 after infection, respectively.

### Immunohistochemistry and double immunohistofluorescence

Peroxidase immunostaining methods were used, as previously described.^30^ IFN-α/β and PRRs were stained in frozen and paraffin-embedded sections, respectively. Sheep polyclonal antibodies to human IFN-α and IFN-β, mouse mAb to human TLR3, and goat polyclonal antibodies to human MDA5 and RIG-I were applied for single immunohistochemistry staining. Normal sheep IgG, irrelevant mouse IgG_1_ κ antibody (MOPC21), and normal goat IgG were used to substitute for the primary layer as a negative control for the staining specificity of sheep polyclonal antibodies, mouse mAb and goat polyclonal antibodies, respectively. Indirect immunofluorescence double staining was used to identify elastase-positive neutrophils and CD68^+^ monocytes/macrophages coexpressing IFN-α/β in frozen sections. We were unable to stain for the type III IFN-λs because no commercially available antibodies were able to stain specifically for these proteins in these biopsy specimens (data not shown).

### Quantification

All quantification was performed on coded slides with the analyst blinded to subject status. Immunostaining intensity for IFN-α, IFN-β, TLR3, MDA5, and RIG-I on bronchial epithelium was quantified by using the hybrid score system.^31^, ^32^ Expression of these proteins was scored based on intensity and fraction of positive cells. Areas of the subepithelium were assessed by using Image 1.5 software. Numbers of subepithelial IFN-α^+^, IFN-β^+^, TLR3^+^, MDA5^+^, and RIG-I^+^ inflammatory cells were counted and expressed as the number of positive cells per square millimeter of subepithelium.

Data for double staining were expressed as the percentage of subepithelial elastase-positive neutrophils or CD68^+^ monocytes/macrophages that were also positive for IFN-α or IFN-β.

### Statistical analysis

Differences between baseline and infection within groups were assessed by using the Wilcoxon matched-pairs test. Differences between groups were analyzed by using the Mann-Whitney *U* test. Spearman rank correlation was used to test associations between staining scores/cell numbers and physiologic/clinical data. A *P* value of less than .05 was considered statistically significant.

Further details of the methods used in this study are provided in the Methods section in this article's Online Repository at www.jacionline.org.

## Results

### Bronchial epithelial IFN-α/β protein expression is deficient in asthmatic patients *in vivo* and is related to rhinovirus-induced clinical illness severity

**Fig 1:**
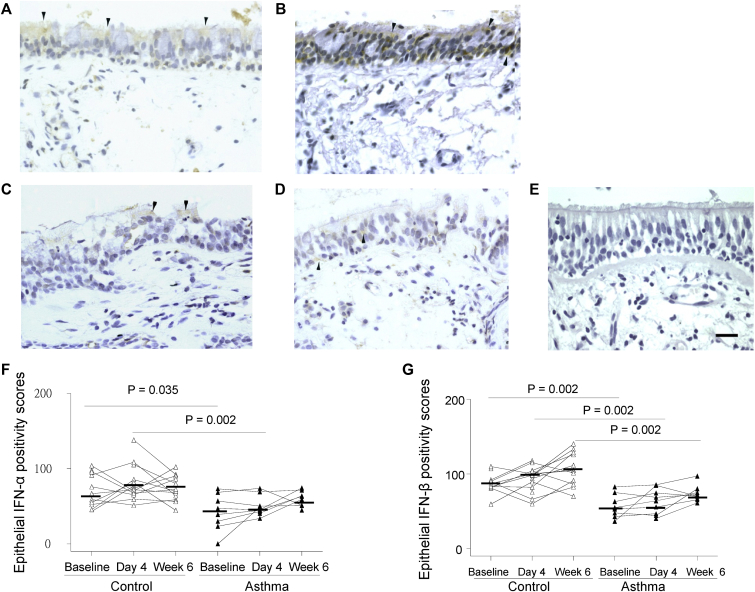
Bronchial epithelial IFN-α and IFN-β protein staining is deficient in asthmatic patients *in vivo*. Immunohistochemistry-stained cells are seen as yellow/brown positivity. **A-D,** A control subject at baseline shows weak (nongoblet) epithelial staining *(arrowheads)* for IFN-α (Fig 1, *A*) and IFN-β (Fig 1, *B*), and an asthmatic patient at baseline demonstrates faint staining *(arrowheads)* in some epithelial cells for IFN-α (Fig 1, *C*) and IFN-β (Fig 1, *D*). **E,** Negative control (normal sheep IgG as primary antibody) shows an absence of signal (*internal scale bar* = 20 μm for all). **F** and **G,** Dot graphs show scores of epithelial staining intensity for IFN-α (Fig 1, *F*) and IFN-β (Fig 1, *G*) in bronchial biopsy specimens of control subjects and asthmatic patients at baseline and day 4 and week 6 after infection. *Triangles* show individual scores, and *horizontal bars* show median values (Wilcoxon matched-pairs test and Mann-Whitney *U* test).

Immunostaining demonstrated weak positivity for both IFN-α and IFN-β in (nongoblet) epithelial cells of control subjects (Fig 1, *A* and *B*) and faint or absent IFN-α and IFN-β staining in asthmatic patients (Fig 1, *C* and *D*). Quantified scores of IFN-α and IFN-β staining intensity were significantly lower in asthmatic patients at baseline (*P* = .035 and .002) and day 4 after infection (*P* = .002 for both; Fig 1, *F* and *G*) and IFN-β scores were also lower at week 6 (*P* = .002; Fig 1, *G*) compared with the same time points in control subjects. There was no significant increase in epithelial staining for IFN-α or IFN-β from baseline to day 4 after infection.

**Fig 2:**
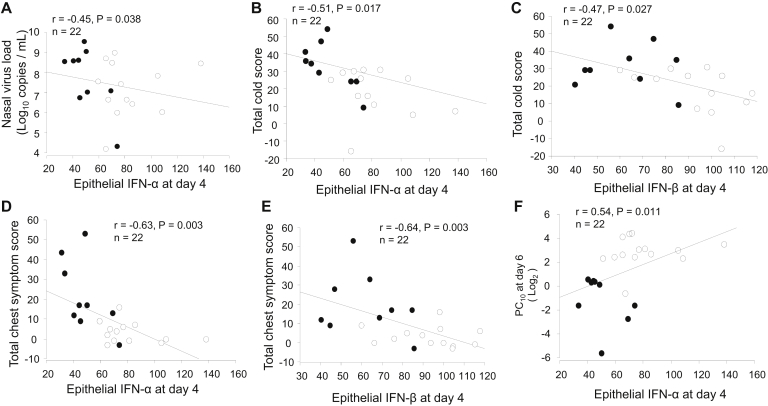

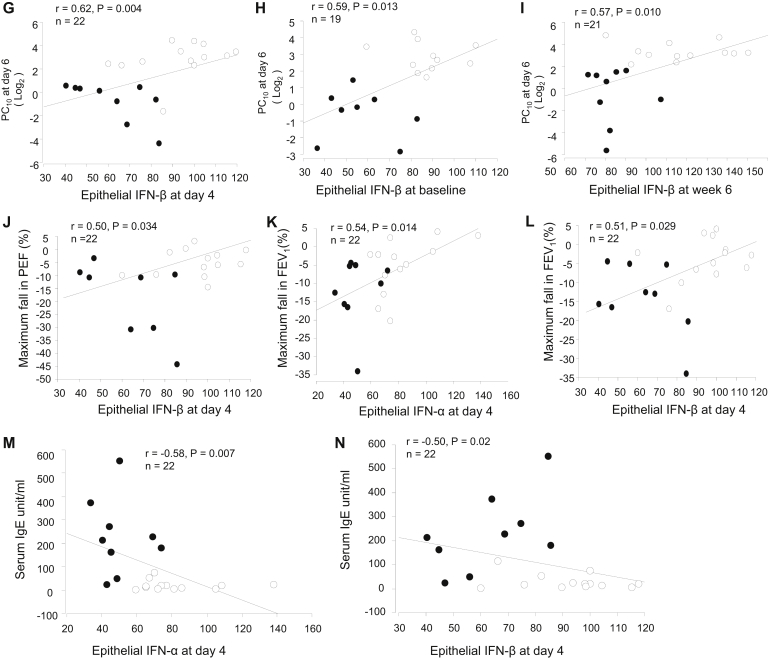
Weaker epithelial IFN-α or IFN-β staining at day 4 after infection is associated with greater viral load and clinical illness severity during rhinovirus infection and with greater baseline serum IgE levels. In all subjects taken together, correlations between nasal viral load and scores of epithelial IFN-α positivity at day 4 **(A)**; between total cold (**B** and **C**) and total chest (**D** and **E**) symptom scores (summed daily scores on days 0-14) after the RV16 infection period and scores of epithelial IFN-α and IFN-β at day 4, respectively; between PC_10_ histamine at day 6 and scores of epithelial IFN-α **(F)** and IFN-β **(G)** at day 4, IFN-β at baseline **(H)** and IFN-β at week 6 **(I)**, respectively; between maximum decrease in PEF (as a percentage) on days 0 to 14 after infection and epithelial IFN-β at day 4 **(J)**; between maximum decrease in FEV_1_ (as a percentage) and scores of epithelial IFN-α **(K)** and IFN-β **(L)** positivity at day 4, respectively; between baseline serum IgE and scores of epithelial IFN-α **(M)** and IFN-β **(N)** positivity at day 4, respectively, are shown. *Solid circles*, Asthmatic patients; *open circles*, control subjects. Spearman rank correlation was used.

**Fig E1:**
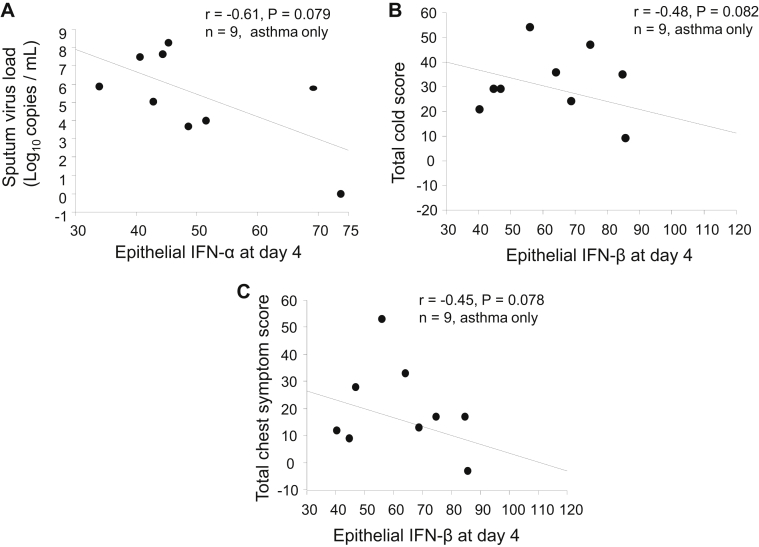
In asthmatic patients only, there is a trend toward correlations between sputum viral load and scores of epithelial IFN-α positivity at day 4 **(A)** and between total cold **(B)** and total chest **(C)** symptom scores (summed daily scores on days 0-14) after the RV16 infection period and scores of epithelial IFN-β at day 4, respectively (Spearman rank correlation).

We next examined whether epithelial staining for IFN-α/β was related to clinical outcomes after rhinovirus infection. In all subjects taken together, lower bronchial epithelial IFN-α staining scores at day 4 after infection were associated with greater viral load in nasal lavage fluid (*r* = −0.45, *P* = .038; Fig 2, *A*). Lower IFN-α and IFN-β scores at day 4 correlated with more severe cold symptom scores (*r* = −0.51 and −0.47, *P* = .017 and .027; Fig 2, *B* and *C*) and chest symptom scores (*r* = −0.63 and −0.64, *P* = .003 for both; Fig 2, *D* and *E*) recorded during the postinfection period and worse AHR (lower histamine PC_10_ at day 6; *r* = 0.54 and 0.62, *P* = .011 and .004; Fig 2, *F* and *G*). Those with weaker epithelial IFN-β staining at both baseline and week 6 after infection also correlated with lower PC_10_ values at baseline (*r* = 0.59, *P* = .013; Fig 2, *H*) and week 6 (*r* = 0.57, *P* = .010; Fig 2, *I*), respectively. Lower bronchial epithelial IFN-β scores at day 4 correlated with larger reductions in PEF (*r* = 0.50, *P* = .034; Fig 2, *J*) during infection. Lower IFN-α and IFN-β scores at day 4 were associated with greater reductions in FEV_1_ (*r* = 0.54 and 0.51, *P* = .014 and .029; Fig 2, *K* and *L*) during infection and also correlated with higher levels of total baseline serum IgE (*r* = −0.58, *P* = .007 and *r* = −0.50, *P* = .02; Fig 2, *M* and *N*). When asthmatic patients and control subjects were analyzed separately, we found a trend toward correlations in asthmatic patients only (ie, *r* = −0.61, *P* = .079 for epithelial IFN-α and sputum viral load [see Fig E1, *A*, in this article's Online Repository at www.jacionline.org]; *r* = −0.48, *P* = .082 for epithelial IFN-β and total cold scores [see Fig E1, *B*]; and *r* = −0.45, *P* = .078 for epithelial IFN-β and total chest symptom scores [see Fig E1, *C*]).

### Bronchial epithelial PRR protein expression was not deficient in asthmatic patients, and TLR3 and MDA5 were induced by rhinovirus infection *in vivo*

**Fig 3:**
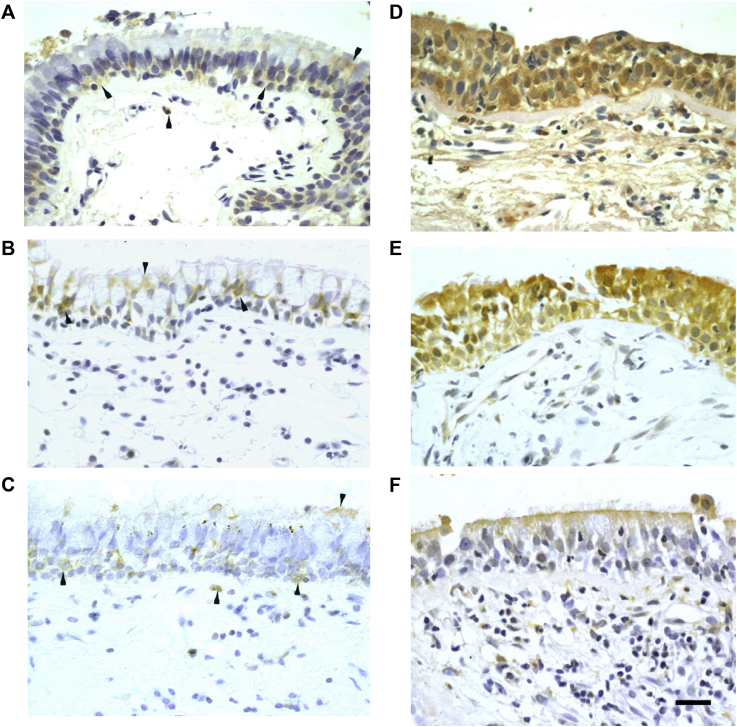

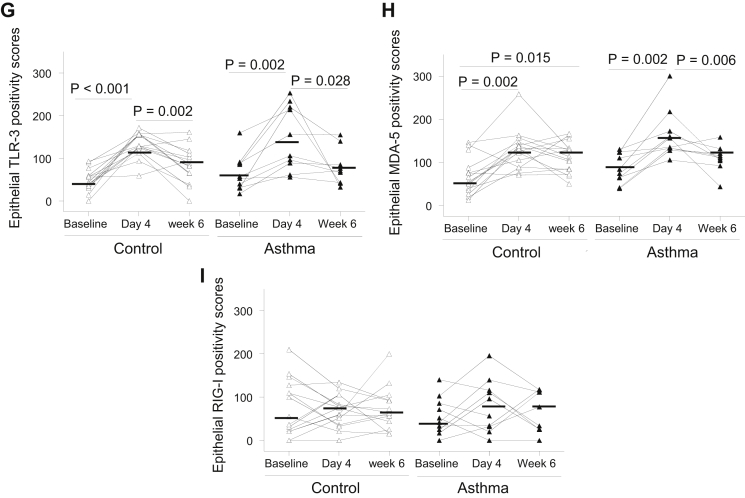
Bronchial epithelial TLR3, MDA5, and RIG-I protein staining is not deficient in asthmatic patients and is increased by rhinovirus infection *in vivo*. Immunohistochemistry-stained cells are seen as yellow/brown positivity. **A-F,** An asthmatic patient shows weak epithelial staining *(arrowheads)* and a few subepithelial positive cells *(arrowheads)* at baseline for TLR3 (Fig 3, *A*), MDA5 (Fig 3, *B*), and RIG-I (Fig 3, *C*) and increased epithelial staining intensity and numbers of subepithelial positive cells at day 4 after infection for TLR3 (Fig 3, *D*), MDA5 (Fig 3, *E*), and RIG-I (Fig 3, *F*; internal scale bar = 20 μm for all). **G-I,** Dot graphs show scores of epithelial staining intensity for TLR3 (Fig 3, *G*), MDA5 (Fig 3, *H*), and RIG-I (Fig 3, *I*) in bronchial biopsy specimens of control subjects and asthmatic patients at baseline and day 4 and week 6 after infection. *Triangles* show individual scores, and *horizontal bars* show median values (Wilcoxon matched-pairs test and Mann-Whitney *U* test).

To determine whether the bronchial epithelial interferon deficiency in asthmatic patients can be explained by reduced baseline PRR expression, we quantified staining for these proteins in bronchial epithelium *in vivo*. Representative photographs for PRR immunostaining in asthmatic patients show weak positivity for TLR3, MDA5, and RIG-I on surface epithelial cells at baseline (Fig 3, *A-C*) and increased strong staining for TLR3 and MDA5 (Fig 3, *D* and *E*) and weak/moderate staining for RIG-I (Fig 3, *E*) on rhinovirus infection. The quantitative scores of epithelial TLR3, MDA5, and RIG-I staining intensity at baseline were not different in asthmatic patients compared with those in control subjects (Fig 3, *G-I*).

Previously, we reported that bronchial epithelial TLR3, MDA5, and RIG-I expression was induced by rhinovirus infection *in vitro*.^15^, ^26^, ^33^ Therefore we determined whether rhinovirus infection could induce these proteins in bronchial epithelium *in vivo*. Scores of epithelial TLR3 and MDA5 staining intensity were increased significantly from baseline values to day 4 after infection in both control subjects (*P* < .001 and *P* = .002, respectively; Fig 3, *G* and *H*) and asthmatic patients (*P* = .002 for both). Scores of epithelial MDA5 positivity remained significantly greater at week 6 after infection than at baseline in control subjects (*P* = .015; Fig 3, *H*). In contrast, at week 6, TLR3 levels in both control subjects and asthmatic patients (*P* = .002 and .028; Fig 3, *G*) and MDA5 levels in asthmatic patients had returned to baseline levels (*P* = .006; Fig 3, *H*). There was no significant induction detected for RIG-I (Fig 3, *I*).

### Total numbers of subepithelial IFN-α/β^+^ and PRR^+^ cells in asthmatic patients were not deficient at baseline, and PRRs were induced by rhinovirus infection *in vivo*

**Fig 4:**
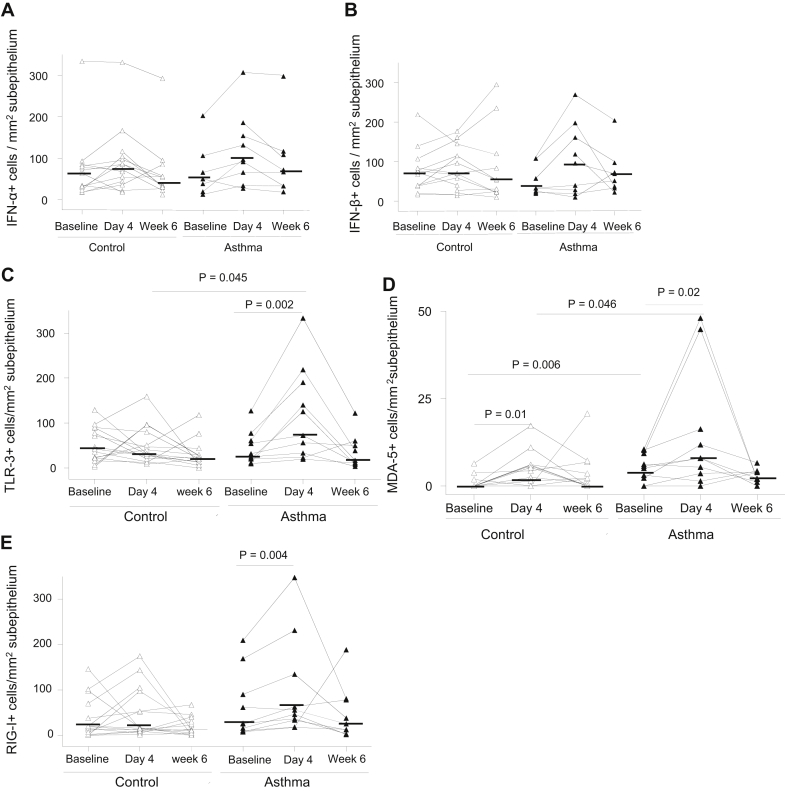
Numbers of subepithelial TLR3, MDA5, and RIG-I protein–expressing inflammatory cells in asthmatic patients are induced in response to rhinovirus infection *in vivo*. Counts for subepithelial IFN-α^+^**(A)**, IFN-β^+^**(B)**, TLR3^+^**(C)**, MDA5^+^**(D)**, and RIG-I^+^**(E)** cells in bronchial biopsy specimens of control subjects and asthmatic patients at baseline and day 4 and week 6 after infection are shown. Data are expressed as numbers of positive cells per square millimeter of subepithelium. *Triangles* show individual counts, and *horizontal bars* show median values (Wilcoxon matched-pairs test and Mann-Whitney *U* test).

Numbers of subepithelial IFN-α^+^ and IFN-β^+^ cells were not significantly different between asthmatic patients and control subjects at baseline, and there were no statistically significant increases in their numbers during infection (Fig 4, *A* and *B*).

Numbers of subepithelial TLR3^+^ and RIG-I^+^ cells at baseline were not significantly different between asthmatic patients and control subjects (Fig 4, *C* and *E*). MDA5^+^ cells at baseline were significantly more numerous in asthmatic patients compared with control subjects (*P* = .006; Fig 4, *D*). Thus there was no evidence of deficient subepithelial PRR-expressing cells at baseline in asthmatic patients.

Numbers of subepithelial TLR3^+^, MDA5^+^, and RIG-I^+^ cells were significantly increased on day 4 after infection in asthmatic patients (*P* = .002, .02, and .004, respectively; Fig 4, *C-E*). MDA5^+^ cell numbers were also increased on day 4 in control subjects (*P* = .01; Fig 4, *D*). Numbers of subepithelial TLR3^+^ and MDA5^+^ cells on day 4 in asthmatic patients were significantly greater compared with those in control subjects on day 4 (*P* = .045 and .046, respectively; Fig 4, *C* and *D*).

### Subepithelial IFN-α^+^, TLR3^+^, and RIG-I^+^ cells during infection are related to viral load and rhinovirus-induced clinical illness severity

**Fig 5:**
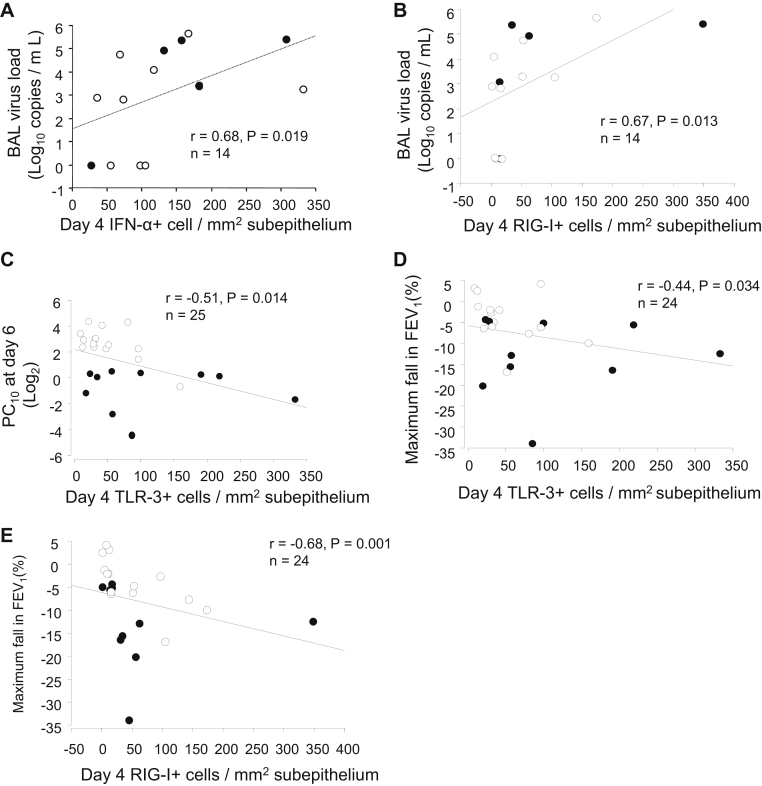
Subepithelial IFN-α and PRR responses at day 4 after infection are associated with greater viral load and AHR and reductions in lung function during infection. Relations between numbers of subepithelial IFN-α^+^**(A)** and RIG-I^+^**(B)** cells at day 4 after infection and BAL fluid viral load, between PC_10_ histamine at day 6 and counts of subepithelial TLR3^+^ cells at day 4 **(C)**, and between maximum decrease in FEV_1_ (as a percentage) on days 0 to 14 after infection and counts of subepithelial TLR3^+^**(D)** and RIG-I^+^**(E)** cells at day 4 are shown. *Solid circles*, Asthmatic patients; *open circles*, control subjects. Spearman rank correlation was used.

To determine whether numbers of subepithelial interferon-positive and PRR^+^ cells were related to protection from disease or to increased disease severity, we examined the association between numbers of positive cells at baseline and during infection to viral load and clinical outcomes. In all subjects taken together, numbers of subepithelial interferon-positive and PRR^+^ cells at baseline were not significantly related to viral load or any clinical outcome during infection (data not shown). However, during acute infection, in all subjects taken together, BAL fluid viral load was significantly associated with numbers of subepithelial IFN-α^+^ (*r* = 0.68, *P* = .019; Fig 5, *A*) and RIG-I^+^ (*r* = 0.67, *P* = .013; Fig 5, *B*) cells. Similarly, greater numbers of subepithelial TLR3^+^ cells during infection were associated with worse rhinovirus-induced AHR (lower day 6 histamine PC_10_ values; *r* = −0.51, *P* = .014; Fig 5, *C*) and reductions in FEV_1_ (*r* = −0.44, *P* = .034; Fig 5, *D*), as were numbers of RIG-I^+^ cells (*r* = −0.68, *P* = .001; Fig 5, *E*). Thus greater numbers of subepithelial interferon-positive/PRR^+^ cells during infection were associated with greater viral load and increased clinical illness severity.

### Numbers of type I interferon–producing monocytes/macrophages were deficient during rhinovirus infection in asthmatic patients

Because type I interferon–producing monocytes/macrophages have potent antiviral activity^34^ and many of the interferon-positive cells in single-stained biopsy specimens rather surprisingly looked morphologically like neutrophils, we next used double-immunofluorescence labeling to determine the numbers of these cell types producing type I interferons during infection.

**Fig 6:**
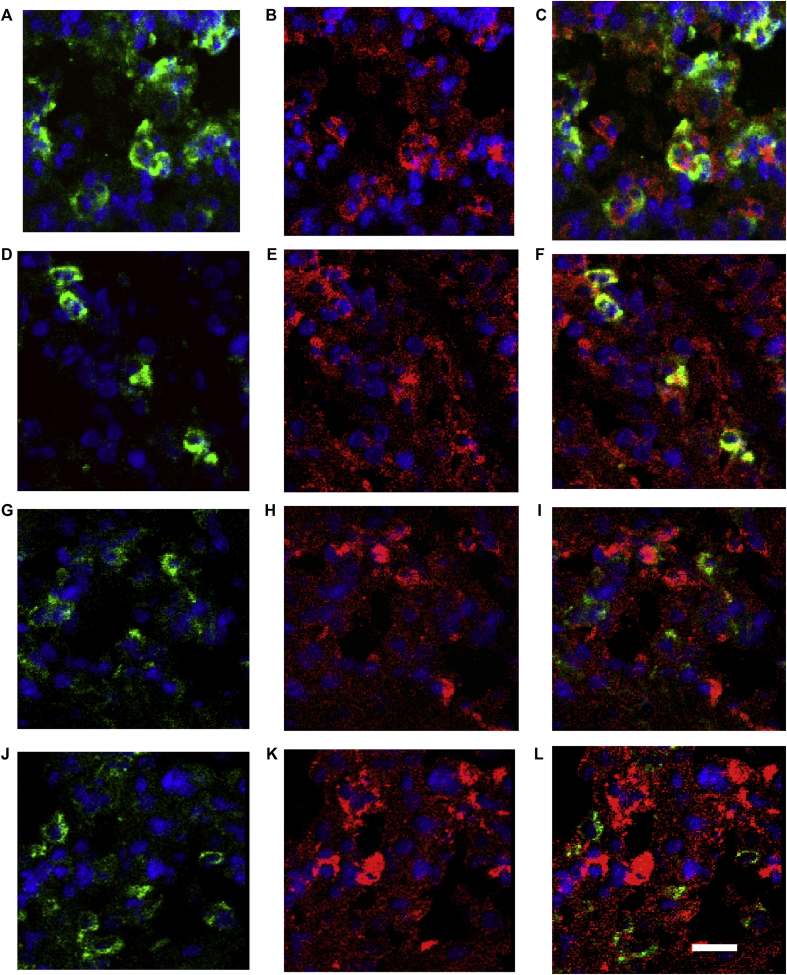

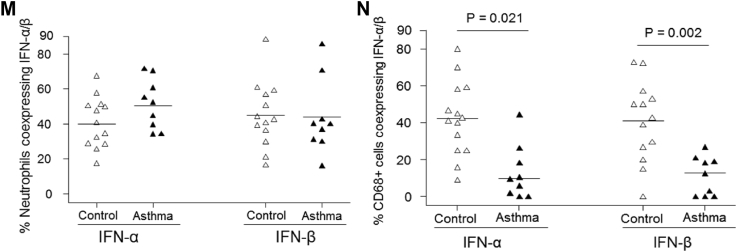
Numbers of subepithelial type I interferon–producing monocytes/macrophages, but not neutrophils, are deficient during rhinovirus infection in asthmatic patients. Results of double-immunofluorescence staining to demonstrate colocalization of neutrophils and CD68^+^ monocytes/macrophages (green) with IFN-α and IFN-β (red) in a bronchial biopsy specimen from an asthmatic patient at day 4 after infection are shown. Elastase-positive neutrophils (**A** and **D**) and CD68^+^ monocytes/macrophages (**G** and **J**) are shown by using fluorescein isothiocyanate green fluorescence, and IFN-α (**B** and **H**) and IFN-β (**E** and **K**) immunopositivity are shown by using Texas Red fluorescence. Coexpression is seen as yellow fluorescence in each case in neutrophils/IFN-α **(C)** and neutrophils/IFN-β **(F)**. However, there are faint or no yellow fluorescence double-labeled cells for CD68/IFN-α **(I)** and CD68/IFN-β **(L)**. *Internal scale bars* = 10 μm for all. Nuclei are counterstained blue with 4′-6-diamidino-2-pheynlindole dihydrochloride. Counting data of double-immunofluorescence staining are shown in graphs of percentages of neutrophils **(M)** and CD68^+^ monocytes/macrophages coexpressing **(N)** IFN-α and IFN-β. *Triangles* show individual percentages, and *horizontal bars* show median values (Mann-Whitney *U* test).

Double-immunofluorescence labeling revealed that neutrophils coexpressed IFN-α (Fig 6, *A-C*) and IFN-β (Fig 6, *D-F*), and most of the monocytes/macrophages were negative for IFN-α (Fig 6, *G-I*) and IFN-β (Fig 6, *J-L*) in asthmatic patients. Counting data demonstrated that as many as approximately 40% to 50% of neutrophils were IFN-α^+^ or IFN-β^+^, and there were no differences in percentages of neutrophils expressing IFN-α and IFN-β between the asthma and control groups (Fig 6, *M*). Similarly, in control subjects approximately 40% of monocytes/macrophages were IFN-α^+^ or IFN-β^+^; in contrast, these frequencies were significantly reduced in asthmatic patients: median of 10% (range, 0% to 44%) for IFN-α and 18% (range, 0% to 30%) for IFN-β (*P* = .021 and .002 respectively; Fig 6, *N*). Thus we observed a striking deficiency in type I interferon–producing monocytes/macrophages in asthmatic patients during rhinovirus infection.

## Discussion

We report that IFN-α and IFN-β protein expression in bronchial epithelium and subepithelial monocytes/macrophages is deficient in asthmatic patients *in vivo*. This epithelial deficiency is related to greater viral load and clinical illness severity (including symptoms, AHR, and lung function) on rhinovirus infection and to higher levels of baseline total serum IgE. Bronchial epithelial interferon deficiency did not appear related to impaired PRR expression because baseline expression of epithelial TLR3, MDA5, and RIG-I in asthmatic patients *in vivo* was not deficient, and TLR3 and MDA5 levels were induced in response to rhinovirus infection. We detected that increases in subepithelial PRR-expressing cells were related to greater viral load and illness severity on rhinovirus infection. Somewhat surprisingly, we also report that neutrophils were a major cell type expressing interferons during rhinovirus infection.

Type I interferons play an essential role in innate antiviral immune responses. Previous *ex vivo* studies revealed that bronchial epithelial cells from asthmatic patients produce less IFN-β^12^ than those from control subjects on exposure to rhinovirus and that this is accompanied by increased viral replication.^12^, ^15^ Others have also demonstrated that rhinovirus-induced IFN-α and IFN-β protein *ex vivo* is delayed and deficient in BAL fluid cells^14^, ^15^ and PBMCs^35^ in asthmatic patients. However, some studies investigating innate interferon responses in asthmatic patients have not found evidence of deficiency, as recently reviewed.^36^ Therefore whether deficient type I interferons are present in the bronchial epithelium of patients with asthma *in vivo* and whether such deficiency is related to clinical outcomes in response to rhinovirus infection was unknown.

We report that in asthmatic patients bronchial epithelium produced less IFN-α and IFN-β at both baseline and day 4 after infection and less IFN-β at 6 weeks compared with the same time points in the control subjects. One would expect epithelial type I interferon expression to be induced during rhinovirus infection *in vivo*; however, we did not observe this at the single day 4 time point studied during infection. A previous study *in vitro* reported that RV16 infection induces IFN-α.2 mRNA expression in human primary bronchial epithelial cells at 8 and 24 hours and IFN-β at only 24 hours.^36^ Bronchial epithelial cells obtained from asthmatic patients and healthy control subjects were cultured *ex vivo* and showed that IFN-β mRNA expression was increased only at 48 hours in both groups in response to RV16 infection.^17^ Therefore it is likely that the time point of day 4 after infection sampled in our study was too late to detect significant epithelial IFN-α/β induction. However, we observed a significant induction of epithelial interferon-induced genes/proteins, TLR3, and MDA5, in both subject groups at day 4. These data are consistent with bronchial epithelial interferons having been induced before day 4. This interpretation is supported by our recent observations in a similar study that type I and III interferons were not induced in BAL fluid on day 4, whereas the interferon-induced proteins CXCL10 and CXCL11 were already significantly induced.^37^ Furthermore, we believe that the immune status of the bronchial epithelium at baseline (ie, before RV16 infection) would be similar to that at 6 weeks after infection. Thus the interferon expression levels we observed at week 6 had already returned to baseline levels.

We also demonstrated that deficient epithelial IFN-α and IFN-β staining scores during rhinovirus infection were significantly associated with greater viral loads and more severe upper and lower respiratory tract symptoms, increases in AHR, and reductions in FEV_1_ and PEF after rhinovirus infection *in vivo*. Our findings suggest that greater bronchial epithelial IFN-α/β expression might be associated with protection against illness severity induced by rhinovirus infection.

Some studies have reported associations between *ex vivo* interferon deficiency and markers of underlying asthma severity,^14^, ^15^, ^16^ and a recent clinical trial showed that worsening of asthma symptoms in response to colds and responses to IFN-β therapy were restricted to moderate or severe underlying asthma.^38^ Our data add further evidence that interferon deficiency is present *in vivo* in the bronchial epithelium in asthmatic patients, even in subjects with normal lung function and treated only with as-required bronchodilators. Unfortunately, asthma control was not assessed in these subjects, and therefore we were unable to relate interferon expression to asthma control. However, we found that interferon deficiency was related to greater baseline serum total IgE levels. These data are consistent with other reports relating interferon deficiency to IgE levels.^27^, ^28^ Further studies of interferon deficiency and response to interferon treatment are required across a range of asthma severities to enable better understanding of these relationships.

Previously, we reported that *ex vivo* study of IFN-β– and IFN-λ–deficient bronchial epithelial cells in patients with severe asthma, baseline expression of TLR3 mRNA, and rhinovirus induction of TLR3, MDA5, and RIG-I mRNA were impaired.^15^ Thus a possible mechanism underlying deficient/delayed interferon responses in asthmatic patients is that interferon induction can be impaired as a result of reduced baseline or virus-induced expression of PRRs, which detect viral infections and induce interferons. We demonstrate here that *in vivo* expression of TLR3, MDA5, and RIG-I protein at baseline and viral induction of TLR3 and MDA5 protein were not impaired in bronchial epithelium in which IFN-α and IFN-β expression was deficient. Our *in vivo* findings extend previous observations that have hitherto only been reported *ex vivo* and indicate a role for PRR expression/induction in interferon deficiency in asthmatic patients.

We recognize from recent literature reports that other TLRs, including TLR2, TLR7, and TLR8, mediate responses to rhinoviruses.^39^, ^40^, ^41^, ^42^ However, at the time our studies were planned in 2007, the major PRRs believed to recognize viral double-stranded RNA and induce type I interferon production in bronchial epithelial cells were TLR3, MDA5, and RIG-I,^26^ hence our focus on these molecules.

Our investigations in the subepithelium, where inflammatory cell recruitment occurs in response to rhinovirus infection,^11^ are more difficult to interpret. Although numbers of subepithelial IFN-α/β^+^ cells were not significantly increased from baseline on day 4, we found that numbers of subepithelial IFN-α^+^ cells on day 4 after infection were associated significantly with BAL viral load. These data would be consistent with greater numbers of subepithelial IFN-α/β–producing cells being recruited in subjects with greater virus loads.

We found that total numbers of subepithelial TLR3^+^, MDA5^+^, and RIG-I^+^ cells were each induced by rhinovirus infection in asthmatic patients *in vivo*, and numbers of TLR3^+^ and MDA5^+^ cells during infection were increased in asthmatic patients compared with control subjects. Numbers of subepithelial TLR3^+^ and RIG-I^+^ cells during infection were related to viral load and rhinovirus-induced clinical illness severity *in vivo*. Functional studies will be required to determine what roles these PRR^+^ cells play in the airway mucosa of asthmatic patients in response to rhinovirus infection.

Our double-staining studies revealed that the frequencies of subepithelial type I interferon–producing monocytes/macrophages were strikingly deficient during rhinovirus infection in asthmatic patients. It is likely that this novel finding in asthmatic patients is functionally important because type I interferon–producing monocytes/macrophages have been shown recently to have potent antiviral activity and to control viral respiratory tract infection and lessen disease severity.^34^ Double staining also revealed that around 40% of subepithelial neutrophils expressed type I interferons during infection. Epithelial cells, monocytes/macrophages,^36^ and dendritic cells^27^ were believed to be the most important cell types producing type I/III interferons in response to viral respiratory tract infections, whereas neutrophils have been reported not to produce type I interferons.^43^ However, other studies reported interferon production by a subgroup of neutrophils in patients with systemic lupus erythematosus^44^ and after granulocyte colony-stimulating factor stimulation.^45^ Further studies are needed to investigate the role of type I/III interferon–producing neutrophils during viral respiratory tract infection.

Because of the invasive nature of bronchoscopic sampling, we were restricted to a single day 4 time point during acute infection. We did not observe interferon induction by rhinovirus infection in the bronchial epithelium or subepithelium *in vivo* at this time point. Further limitations were the relatively small numbers of subjects studied, which might have limited our statistical power to detect potentially significant findings (ie, significant correlations were not found in the asthma group alone), probably because only 10 asthmatic patients were available to study. Additionally, the asthmatic patients had a sex bias, with 8 of 10 being female, whereas there was no sex bias in the control subjects. We are not aware of differences in antiviral immunity among different sexes, but this disparity could have introduced bias.

In conclusion, we have demonstrated IFN-α/β deficiency in the airway epithelium and subepithelial monocytes/macrophages of asthmatic patients *in vivo*, and the epithelial interferon deficiency was associated with greater viral load and illness severity on rhinovirus infection. Interestingly, subepithelial neutrophils were the source of IFN-α/β in asthmatic patients during infection. Interferon deficiency was not accompanied by deficient expression or rhinovirus induction of PRRs. Rhinovirus infection induced more subepithelial PRR^+^ cells in asthmatic patients, with higher levels of TLR3 and RIG-I expression linked to greater viral load and worse clinical outcomes, suggesting such responses are markers of severity of infection. These collective observations add to our understanding of the interplay between innate antiviral and proinflammatory immune responses and to a greater appreciation of the role of type I interferon responses during rhinovirus-induced asthma exacerbations.

**Key messages**

• IFN-α and IFN-β protein expression in the bronchial epithelium and subepithelial monocytes/macrophages is deficient in asthmatic patients *in vivo*. This epithelial deficiency is related to greater viral load and clinical illness severity on rhinovirus infection and to greater levels of baseline total serum IgE.
• Bronchial epithelial interferon deficiency did not appear related to impaired PRR expression. Mucosal expression of TLR3, MDA5, and RIG-I in asthmatic patients *in vivo* was induced in response to rhinovirus infection. Increased numbers of subepithelial IFN-α^+^, TLR3^+^, and RIG-I^+^ cells during infection are related to greater viral load/illness severity.
• The present findings add to our understanding of the role of type I interferon and PRR responses during rhinovirus-induced asthma exacerbations.

## Figures and Tables

**Table E1:** Baseline individual demographic data

Subjects	Sex (M/F)	Age (y)	Skin prick test response positivity (total scores)	Serum IgE (U/mL)	Histamine PC_20_ (mg/mL)	FEV_1_ (% predicted)	Treatment
Asthmatic patients							
1	F	19	18	50	3.5	104	β_2_
2	M	28	19	553	0.2	98	β_2_
3	F	24	11	180	1.1	114	β_2_
4	F	19	13	271	3.0	100	β_2_
5	F	24	9	229	0.2	83	β_2_
6	M	19	0	162	2.8	105	β_2_
7	F	22	5	212	7.4	118	β_2_
8	F	21	27	374	1.4	104	β_2_
9	F	22	7	355	3.9	101	β_2_
10	F	31	15	25	3.7	136	β_2_
Mean		23	12∗	241∗	2.7∗	106	
SEM		1.4	2.5	49.8	0.7	4.4	
Control subjects							
11	M	28	0	24	32	101	—
12	M	29	0	115	26	90	—
13	F	21	0	22	32	103	—
14	M	35	0	2	9	99	—
15	F	31	0	7	8	122	—
16	M	53	0	76	32	110	—
17	M	24	0	5	17	99	—
18	M	19	0	19	10	99	—
19	F	24	0	22	32	99	—
20	F	28	0	14	16	136	—
21	M	18	0	6	12	109	—
22	F	23	0	11	16	85	—
23	F	31	0	6	32	116	—
24	F	18	0	16	16	105	—
25	M	22	0	54	10	95	—
Mean		27	0	27	20	104	
SEM		2.3	0	8.0	2.5	3.3	

∗ *P* < .0001 versus controls (Student *t* test).

## Methods

### Subjects

Here we extended our investigation of 25 previously reported nonsmokers.^E1^ Those subjects were human RV16 neutralizing antibody seronegative, and the group comprised 10 patients with atopic asthma and 15 healthy nonatopic control subjects (Table E1). Clinical and atopic status were defined by questionnaire, skin prick testing, serum IgE measurement, and lung function testing, including PEF, FEV_1_, and forced vital capacity.^E1^ Four of 10 asthmatic patients had pollen sensitization with seasonal rhinitis. These subjects were studied outside their allergen seasons when they had been asymptomatic for at least 6 weeks. The healthy control subjects were taking no medication. Asthmatic patients were prescribed inhaled short-acting β_2_-agonists only. Subjects were free of common cold symptoms for at least 6 weeks before the study's start.^E1^

### Histamine challenge

Bronchial reactivity was measured by means of histamine challenge at baseline and on day 6 after infection and convalescence. For bronchodilator reversibility, subjects undergoing sputum induction and histamine challenge used a Vitalograph Dry Wedge Bellows Spirometer (Vitalograph, Lenexa, Kan). Histamine challenge (maximum concentration, 32 mg/mL) was performed according to guidelines by using the 2-minute tidal breathing method.^E2^ The asthma group was required to have a PC_20_ value of less than 8 mg/mL, and the control group had a PC_20_ value of greater than 8 mg/mL. The FEV_1_ PC_20_ value was calculated,^E2^ and because 8 control subjects did not achieve a 20% reduction, a PC_10_ value was also calculated for both groups.

### Skin prick testing and serum IgE measurement

Atopy was determined by using skin prick testing to common aeroallergens: 6-grass-pollen mix, house dust mite, cat, dog, *Aspergillus fumigatus*, *Cladosporium herbarum*, *Alternaria alternata*, and birch, three tree, and nettle pollen (ALK-Abelló, Hørsholm, Denmark). One wheal 3 mm larger than that elicited by the negative control or a total IgE level of greater than 110 U/mL was considered diagnostic of atopy.^E1^

### Virologic confirmation of RV16 infection

RV16 infection was confirmed by at least 1 of the following: positive nasal lavage standard or quantitative PCR results for rhinovirus and positive culture of RV16 from induced sputum and BAL fluid. Seroconversion was defined as a titer of serum neutralizing antibodies to RV16 of at least 1:4 at 6 weeks.^E1^

### Diary cards for symptom scores and spirometric recording

Symptom assessment was performed with daily diary cards for 2 weeks before, during, and 6 weeks after infection. The daily cold score was summed from individual scores (sneezing, headache, malaise, chilliness, nasal discharge, nasal obstruction, sore throat, cough, and fever) graded 0 (absent) to 3 (severe). The daily chest score was calculated from symptom scores (cough on waking, wheeze on waking, daytime cough, daytime wheeze, daytime shortness of breath, and nocturnal cough, wheeze, or shortness of breath) graded 0 to 3. The same diary cards recorded medication use and home spirometry (microDL; MicroMedical, Rochester, UK), recording the best of 3 PEF and FEV_1_ measurements.^E1^

### Bronchoscopy and clinical data

Not all frozen biopsy specimens from all 3 bronchoscopy occasions (baseline, day 4, and week 6) obtained from 10 asthmatic patients and 15 healthy subjects met the required standard for inclusion in the study for analysis of epithelial and subepithelial IFN-α/β expression. For example, the baseline frozen biopsy specimens from 4 of 15 control subjects and 2 of 10 asthmatic patients were of inadequate size and were excluded. Consequently, there were only 19 subjects for tests of correlation between baseline epithelial IFN-β and PC_10_ values at day 6. Similarly, only 21 biopsy specimens were suitable for analysis at 6 weeks for correlation between week 6 epithelial IFN-β and PC_10_ values at day 6. In addition, viral load/clinical data were not available for all subjects at all time points; that is, there were only 5 of 10 and 9 of 15 subjects with BAL viral load determined among the asthmatic patients and healthy control subjects (Fig 5, *A* and *B*; n = 14), respectively. Also, there were 14 of 15 subjects with maximum percentage decrease in FEV_1_ available among the control subjects (Fig 5, *D* and *E*; n = 24).

### Immunohistochemistry

IFN-α/β and PRRs were stained in frozen and paraffin-embedded sections, respectively. Peroxidase immunostaining methods were used, as previously described,^E3^ to detect IFN-α, IFN-β, TLR3, RIG-1, and MDA5 protein expression. The following panel of antibodies was applied to tissue sections: sheep polyclonal antibodies to human IFN-α (31100-1) and IFN-β (31400-1; PBL Biomedical Laboratories, Piscataway, NJ), a mouse mAb to human TLR3 (IMG-315A; Imgenex, San Diego, Calif), and goat polyclonal antibodies to human RIG-I (sc-48929) and MDA5 (sc-48031; Santa Cruz Biotechnology, Dallas, Tex). Normal sheep IgG (sc-2765; Santa Cruz Biotechnology), irrelevant mouse IgG_1_ κ antibody (MOPC21; SAB4700724; Sigma-Aldrich, St Louis, Mo), and normal goat IgG (sc-2755) were used to substitute for the primary layer as negative controls for staining specificity of sheep polyclonal antibodies, mouse mAbs, and goat polyclonal antibodies, respectively.

The frozen sections and dewaxed paraffin-embedded sections were incubated with 3% H_2_O_2_ and then blocked with 10% normal rabbit or goat or swine serum in PBS (pH 7.4). Sections were then incubated with anti–IFN-α (1:100), anti–IFN-β (1:50), anti-TLR3 (1:50), anti–RIG-1 (1:150), and anti-MDA5 (1:250) antibodies at room temperature; washed; and incubated with horseradish peroxidase (HRP)–conjugated rabbit anti-sheep (F0163; Dako, Cambridge, United Kingdom) for IFN-α and IFN-β, HRP-conjugated goat anti-mouse (K4001; Dako) for TLR3, and LSAB+System-HRP (K0690, Dako) for RIG-I and MDA5. After a further wash, bound HRP was detected as a dark brown product after incubation with 3, 3-diaminobenzidine. Slides were counterstained with hematoxylin to provide morphologic details and then mounted in DPX mounting medium.

To confirm the staining specificity of the sheep polyclonal antibodies used to detect IFN-α and IFN-β, we tested both mouse mAbs and sheep polyclonal antibodies to human IFN-α and IFN-β in adjacent tissue sections of bronchial biopsy specimens (product number for mouse mAbs: 21100-1 for α and 21400-1 for β; PBL Biomedical Laboratories). We showed that our sheep polyclonal antibodies were as specific as the mouse mAbs because 2 types of the antibodies identified the same positively stained epithelial cells and subepithelial inflammatory cells in 2 adjacent sections of bronchial mucosa. Nonspecific staining background of sheep polyclonal antibodies was minimized by using a standard blocking procedure. The positive signal and background were well contrasted, as shown below in Fig 1, *A-D*. Also, normal sheep IgG was used to substitute for the primary layer as a negative control for the staining specificity of sheep polyclonal antibodies, which were shown as negative in Fig 1, *E*.

### Double immunohistofluorescence

Immunohistofluorescence staining for IFN-α and IFN-β was similarly carried out by using the above antibodies in frozen sections. Primary anti-human antibodies for neutrophils and monocytes/macrophages were mouse anti-neutrophil elastase (M0752) and anti-CD68 (M0876; Dako). Bound antibodies were detected with a combination of the appropriate green fluorescein isothiocyanate– or Texas red–conjugated donkey secondary antisera (Jackson ImmunoResearch, West Grove, Pa). Sections were counterstained with 4′-6-diamidino-2-pheynlindole dihydrochloride. Images were acquired with a Leica TCS-SP confocal microscope with lasers producing excitation wavelengths of 351 to 364 nm, 488 nm, and 568 nm (Leica Microsystems UK, Milton Keynes, United Kingdom).

### Quantification

All quantifications were performed on coded slides with the analyst blind to subject status. The immunostaining intensity for IFN-α, IFN-β, TLR3, MDA5, and RIG-I on bronchial epithelium was quantified by using the hybrid score system.E4, E5 Expression of these proteins was scored based on the intensity and fraction of positive cells. The scored range was from 0 to 4. The intensity score was defined as follows: 0, negative compared with the background or no specific staining; 1, barely detectable staining in the cytoplasm; 2, weak staining distinctly marking the epithelial cytoplasm; 3, moderate staining in the cytoplasm; or 4, strong staining in the cytoplasm. The score was based on the fraction of positive cells (0% to 100%). The total score was calculated by multiplying the intensity score (0-4) and percentage of positive cells in each score, which produce a total score range of 0 to 400, as previously reported.E4, E5 The subepithelial areas, excluding muscles and glands, in bronchial biopsy specimens were assessed by using an Apple Macintosh computer (Apple, Cupertino, Calif) and Image 1.5 software. Numbers of IFN-α^+^, IFN-β^+^, TLR3^+^, MDA5^+^, and RIG-I^+^ inflammatory cells were counted with a Leitz Dialux 20 light microscope (Leitz, Wetzlar, West Germany), working at ×200 magnification and fitted with an eyepiece graticule divided into 100 squares. Two to 3 bronchial biopsy specimens for each biopsy of each subject were measured and counted to take account of within-subject variability. The average of the counts for each subject was used for statistical analysis. Data for bronchial biopsy cell counts were expressed as the number of cut cell profiles with a nucleus visible (ie, positive cells) per square millimeter of subepithelium.

The coefficient of variation for repeat counts of subepithelial inflammatory cells positive for the immune markers by one observer ranged between 5% and 6%.

Counting immunofluorescence double-staining for IFN-α/β with neutrophils or monocytes/macrophages was performed on the images acquired with a Leica TCS-SP confocal microscope with Leica confocal software. Data for double staining were expressed as the percentage of elastase-positive neutrophils or CD68^+^ monocytes/macrophages coexpressing IFN-α or IFN-β in the subepithelial zone of bronchial biopsy specimens.

### Statistical analysis

Statistical analysis was performed with StatView (SAS Institute, Cary, NC) and GraphPad Prism 4 (GraphPad Software, La Jolla, Calif) software. One-way ANOVA, followed by the unpaired Student *t* test, was used for analyses of age, serum IgE, lung function, and histamine PC_20_ data between groups. In respect to cell counts, these data were nonnormally distributed, and differences between groups were assessed first by using the Kruskal-Wallis test, which, if results were significant, was followed by the Wilcoxon matched-pairs test within groups between baseline and infection and the Mann-Whitney *U* test between groups at baseline and infection. The coefficient of variation (ie, SD/mean × 100) was used to express the error of repeat counts. A *P* value of less than .05 was accepted as indicating a significant difference. Spearman rank correlation was used as a test for correlations between the epithelial staining scores/numbers of specific types of inflammatory cells and serum IgE levels plus 8 parameters representing the most important markers of clinical illness severity after experimental rhinovirus infection: viral load (peak nasal lavage: day 3 sputum and day 4 BAL), AHR (day 6 PC_10_ histamine), clinical symptoms (total cold score and total chest score), and airflow obstruction (maximal decrease in PEF and in FEV_1_). Analyses were performed including all subjects with available counts and viral load/clinical data.
